# Barley Seedling Extracts Inhibit RANKL-Induced Differentiation, Fusion, and Maturation of Osteoclasts in the Early-to-Late Stages of Osteoclastogenesis

**DOI:** 10.1155/2017/6072573

**Published:** 2017-05-08

**Authors:** Sik-Won Choi, Shin-Hye Kim, Kwang-Sik Lee, Hyeon Jung Kang, Mi Ja Lee, Kie-In Park, Jin Hwan Lee, Ki Do Park, Woo Duck Seo

**Affiliations:** ^1^Division of Crop Foundation, National Institute of Crop Science (NICS), Rural Development Administration (RDA), Jeonbuk 565-851, Republic of Korea; ^2^Department of Biological Sciences, College of Natural Science, Chonbuk National University, Jeonbuk 561-756, Republic of Korea; ^3^College of Crop Science and Biotechnology, Dankook University, Cheonan 330-714, Republic of Korea; ^4^Division of Research Development and Education, National Institute of Chemical Safety, Ministry of Environment, Daejeon 305-343, Republic of Korea

## Abstract

The number of patients with osteoporosis is increasing worldwide, and a decrease in bone mass is a main risk factor for fracture. The prevention of bone loss is critical for improving the quality of life for patients. However, the long-term use of antiosteoporotic agents is limited due to their side effects. Barley has been traditionally ingested for thousands of years as a safe, natural food with pharmaceutical properties, and its seedling can enhance the biological activity of the medicinal components found in food. This study aimed to clarify the antiresorptive activity of barley seedling and its mode of action. Barley seedling extracts (BSE) dose-dependently inhibited RANKL-induced osteoclast differentiation with alteration of I*κ*B degradation, c-Fos, and NFATc1 molecules in the early-to-middle stages of osteoclastogenesis. In the late phase of osteoclastogenesis, BSE also prevented DC-STAMP and cathepsin K, which are required for cell fusion and bone degradation, such as osteoclast function. In conclusion, barley seedling from natural foods may provide long-term safety and be useful for the prevention or treatment of osteoclast-mediated bone metabolic diseases, including osteoporosis.

## 1. Introduction

Bones are dynamic structures that are continually being formed and resorbed through the constant processes of remodeling and reorganisation. Bone homeostasis is sustained by a tight balance between osteoclast-mediated bone destruction and osteoblast-related bone formation. However, an imbalance of bone homeostasis by the induction of osteoclastic bone destruction or by the reduction of osteoblastic bone formation can lead to a variety of bone metabolic disorders, including osteoporosis, rheumatoid arthritis, and Paget's disease [1–3]. Most bone metabolic disorders induce the activation of osteoclasts; consequently, bone resorption can exceed bone formation and lead to pathological bone-resorbing activity resulting in osteopenic disorders with increased risk of fracture [4, 5]. The economic burdens for hospitalisation, skeletal deformity, and pain due to fractures have become a serious public health issue worldwide [6]. Therefore, protection against bone loss and the risk of bone fracture is an essential means of improving the quality of patients' lives with bone defects.

The differentiation of osteoclasts is a complex multistep process that involves cell differentiation, migration, fusion, and resorption. In bone marrow, osteoclasts are multinucleated giant cells that resorb mineralised tissues; they are differentiated from hematopoietic stem cells by key regulators, such as receptor activator of NF-*κ*B ligand (RANKL) and macrophage colony-stimulating factor (M-CSF) [7]. M-CSF and RANKL trigger the differentiation of osteoclast precursors into mononuclear osteoclasts (preosteoclasts) and increase them to migrate until they attach to the bone matrix. Mononuclear osteoclasts then fuse to form giant multinucleated osteoclasts that, subsequently, relate to bone resorption. In the initial stage, the binding of RANKL to its receptor RANK triggers osteoclast differentiation by the activation of mitogen-activated protein (MAP) kinases and transcription factor, NF-*κ*B [8]. These essential signaling molecules contribute to the activation of c-Fos and nuclear factor of activated T cells, cytoplasmic 1 (NFATc1), which are known to be master regulators for osteoclast differentiation [7, 9, 10]. c-Fos is induced in the early-to-middle stages of osteoclastogenesis and NFATc1 is increased in the middle-to-late phases. These two transcription factors play a critical role in the expression of specific genes, such as tartrate-resistant acid phosphatase (TRAP), osteoclast-associated receptor (OSCAR), dendritic cell-specific transmembrane protein (DC-STAMP), and cathepsin K, which are required for osteoclast differentiation, cell fusion, and maturation. Thus, the regulation of osteoclast formation-mediated molecules is essentially responsible for the degradation of the mineralised matrix during physiological and pathological bone turnover [11].

For thousands of years, plant foods, including vegetables, fruit, wheat, rice, and barley, have been conventionally ingested in many countries around the world due to their nutritional support for the body. Moreover, plant foods contain naturally occurring bioactive components known as phytochemicals. Therefore, plants are a source of safe, healthy foods because they are suitable for long-term use, although the fact that ingesting plants might have therapeutic benefits is clearly not a new concept. Specifically, functional foods, and their bioactive compounds that play a role in improving skeletal health, have received noticeable attention. Recently, a number of studies have reported that functional foods and their phytochemicals prevent bone loss in both female and male osteoporotic animal models, as well as in postmenopausal women [12–15]. Therefore, dietary intake of natural bioactive plant foods is an adaptable habit that may play a key role in reducing the risk of diseases disorders, such as osteoporosis.

The various physiological functions of barley have been reported to exhibit antioxidative, anti-inflammatory, antiobesity, hair-growth stimulation, and cholesterol-lowering activities [16–21]. These studies have attracted considerable research attention focusing on the biological activity of barley, and its evident safety valuation has accelerated the commercial use of barley and its phytochemicals. Furthermore, researchers have shown an interest in developing natural ingredients that can increase the bioactive components in barley. Notably, the barley seedling (BS), grown for about 7 days from barley seed, contains high concentrations of various physiologically active ingredients that enable it to germinate and to protect itself from external attacks. In particular, BS contains policosanols with substantial levels of polyphenol and saponarin as a major flavonoid, which have a variety of biological activities [22, 23]. Accordingly, the pharmaceutical properties of BS have potential roles in the prevention and treatment of disease. Few studies have investigated barley seedling extracts (BSE), and it has not been elucidated as to whether or not BSE has antiresorptive activity. Therefore, we investigated the effect of BSE on RANKL-mediated osteoclast differentiation and the bone-resorbing activity of mature osteoclasts.

## 2. Materials and Methods

### 2.1. Preparation of the Barley Seedling Extracts

Barley (*Hordeum vulgare* L.) was cultivated in 2015 in the experimental field at the National Institute of Crop Science, Rural Development Administration, Jeonbuk, Korea. BS was prepared using the procedure reported in the literature [23]. Barley seeds were washed twice using deionized distilled water and imbibed in water at 18°C for 24 hr. The imbibed seeds were germinated in 65% humidity at 16°C in a normal light cycle (16/8 hr day/night). The germinating seeds and seedlings were harvested in liquid nitrogen 7 days after germination. The collected leaves were freeze-dried immediately after sampling. Prior to obtaining the BSE, the leaves were pulverised to 100 mesh. The masses of all the samples were based on dry weight. To determine the antiosteoporotic activities, the pulverised seeds (10 g) were extracted with 20 ml of the prethanol for 2 days at 4°C in the dark. The crude extracts were filtered through Whatman Number 42 filter paper to remove the sediment. The solvent was evaporated, and the prethanol extracts were obtained from the BS.

### 2.2. Reagents and Antibodies

Mouse soluble RANKL and M-CSF were purchased from R&D Systems (Minneapolis, MN, USA). Penicillin, streptomycin, cell culture medium, and foetal bovine serum (FBS) were purchased from Invitrogen Life Technologies (Carlsbad, CA, USA). Antibodies against NFATc1, actin, and I*κ*B and secondary antibody conjugated to horseradish peroxidase (HRP) were purchased from Santa Cruz Biotechnology (Dallas, TX, USA). All of the other antibodies were obtained from Cell Signaling Technology (Beverly, MA, USA).

### 2.3. Ethics Statement

This study was conducted in strict accordance with the recommendations in the Standard Protocol for Animal Study of Gangnam Severance Hospital Biomedical Center (Permit Number 2016-0238). The protocol (ID Number 0238) was approved by the Institutional Animal Care and Use Committee (IACUC) of Yonsei University College of Medicine. Every effort was made to minimise the number of animals used in the study and minimise their suffering and stress/discomfort.

### 2.4. Preparation of Osteoclast Precursor Cells

All the experiments were carried out as described in a previous study, with modifications [24]. Five-week-old male Imprinting Control Region (ICR) mice (Damul Science Co., Daejeon, Korea) were maintained in a room illuminated daily from 07:00 to 19:00 (12 : 12 hr light/dark cycle), with controlled temperature (23 ± 1°C) and ventilation (10–12 times per hour); humidity was maintained at 55 ± 5% and the animals had free access to a standard animal diet and tap water. Bone marrow cells were obtained from the five-week-old male ICR mice by flushing their femurs and tibias with alpha minimum essential medium- (*α*-MEM-) containing antibiotics (100 units/ml penicillin, 100 *μ*g/ml streptomycin). The bone marrow cells were cultured on culture dishes for 1 day in *α*-MEM containing 10% FBS and M-CSF (10 ng/ml). The nonadherent bone marrow cells were plated into Petri dishes and cultured for 3 days in the presence of M-CSF (30 ng/ml). After the nonadherent cells were washed out, the adherent cells were used as bone marrow-derived macrophages (BMMs).

### 2.5. Osteoclast Cell Culture and Osteoclast Differentiation

The BMMs were maintained in *α*-MEM supplemented with 10% FBS, 100 units/ml penicillin, and 100 *μ*g/ml streptomycin. The medium was changed every 3 days in a humidified atmosphere of 5% CO_2_ at 37°C. To differentiate the osteoclasts from the BMMs, the BMMs (1 × 10^4^ cells/well in a 96-well plate or 3 × 10^5^ cells/well in a 6-well plate) were cultured with M-CSF (30 ng/ml) and RANKL (10 ng/ml) for 4 days, and then the multinucleated osteoclasts were observed.

### 2.6. TRAP Staining and Activity Assay

The mature osteoclasts were visualised using TRAP staining, a biomarker of osteoclast differentiation. Briefly, the multinucleated osteoclasts were fixed with 3.7% formalin for 10 min, permeabilised with 0.1% Triton X-100 for 10 min, and then stained with TRAP solution (Sigma-Aldrich, Saint Louis, MO, USA). The TRAP-positive multinucleated osteoclasts (MNC; nuclear ≥ 3 or nuclear ≥ 10) were counted. To measure TRAP activity, the multinucleated osteoclasts were fixed in 3.7% formalin for 5 min, permeabilised with 0.1% Triton X-100 for 10 min, and then treated with TRAP buffer (100 mM sodium citrate, pH 5.0, 50 mM sodium tartrate) containing 3 mM* p*-nitrophenyl phosphate (Sigma-Aldrich) at 37°C for 5 min. The reaction mixtures in the wells were transferred to new plates containing an equal volume of 0.1 N NaOH, and the optical density values were determined at 405 nm.

### 2.7. Cell Viability Assay

The BMMs were plated in a 96-well plate at a density of 1 × 10^4^ cells/well, in triplicate. After treatment with M-CSF (30 ng/ml) and various concentrations of BSE, the cells were incubated for 3 days, and cell viability was measured using the Cell Counting Kit 8 (CCK-8) according to the manufacturer's protocol. The CCK-8 assay kit was purchased from Dojindo Molecular Technologies (Rockville, MD, USA).

### 2.8. RNA Isolation and Real-Time Polymerase Chain Reaction Analysis

Real-time polymerase chain reaction (PCR) was performed as described previously [25]. The primers were chosen using the online Primer3 design program [26]. The primer sets used in this study are shown in Table 1. Briefly, total RNA was isolated with TRIzol reagent, and the first-strand cDNA was synthesized with the RevertAid First-Strand cDNA Synthesis Kit (Thermo Scientific, Waltham, MA, USA) according to the manufacturer's recommended protocol. SYBR green-based quantitative PCR (qPCR) was performed using the Bio-Rad CFX96 Real-Time PCR Detection System (Hercules, CA, USA) and Topreal qPCR 2x PreMIX (Enzynomics, Daejeon, Korea). All reactions were run in triplicate, and the data were analysed using the 2^−ΔΔCT^ method [27]. Hypoxanthine phosphoribosyltransferase 1 (HPRT1) and glyceraldehyde 3-phosphate dehydrogenase (GAPDH) were used as the internal standard genes. The statistical significance was determined using Student's *t*-test with HPRT1/GAPDH-normalised 2^−ΔΔCT^ values; the differences were considered significant at *P* < 0.05.

### 2.9. Western Blot Analysis

Western blot analysis was performed as described previously [28]. Briefly, the cultured cells were washed with ice-cold phosphate-buffered saline (PBS) and lysed in lysis buffer (50 mM Tris-HCl, 150 mM NaCl, 5 mM EDTA, 1% Triton X-100, 1 mM sodium fluoride, 1 mM sodium vanadate, and 1% deoxycholate) supplemented with protease inhibitors. After centrifugation at 15,000 ×g for 15 min, the protein quantification in the supernatant was determined using the detergent compatible (DC) protein assay kit (Bio-Rad). The quantified proteins were denatured, separated on sodium dodecyl sulphate-polyacrylamide gel electrophoresis (SDS-PAGE) gels, and transferred onto a polyvinylidene difluoride (PVDF) membrane (Merck Millipore, Darmstadt, Germany). After incubation with an antibody, the membranes were developed using SuperSignal West Femto Maximum Sensitivity Substrate (Thermo Scientific) and visualised with the LAS-4000 luminescent image analyser (GE Healthcare Life Sciences, Little Chalfont, UK). Actin was used as a loading control.

### 2.10. Bone Pit Formation Analysis

The mature osteoclasts were prepared by isolating osteoblasts from the calvariae of newborn mice by serial digestion in 0.1% collagenase (Gibco, Paisley, UK), as previously described [29]. The bone marrow cells were isolated as described above. The osteoblasts (3.5 × 10^5^ cells/well) and BMMs (1 × 10^6^ cells/well) were cocultured on a collagen-coated 90 mm dish in the presence of 1*α*,25-dihydroxyvitamin D_3_ (VitD_3_) and prostaglandin E_2_ (PGE_2_) for 6 days. The *α*-MEM complete medium with VitD_3_ and PGE_2_ was changed every 3 days. The cocultured cells were detached from the collagen-coated dishes using 0.1% collagenase and then replated on a bone biomimetic synthetic surface (Corning, NY, USA) in a 24-well plate. After 1 hr, each well was treated with RANKL (10 ng/ml) and BSE for 24 hrs. The cells on these plates were stained for TRAP and photographed under a light microscope at 10x magnification. To observe the resorption pits, the slides were washed with PBS and treated with 5% sodium hypochlorite for 5 min. After the plate was washed with PBS and dried, it was photographed under a light microscope. Quantification of the resorbed areas was performed using the ImageJ program.

### 2.11. Statistical Analysis

**Figure 1:**
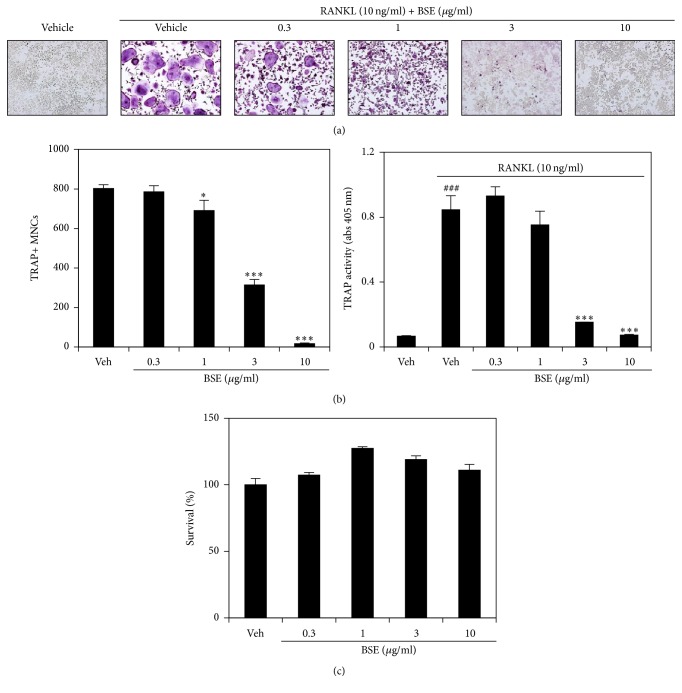
BSE impairs RANKL-induced osteoclast differentiation. (a) The BMMs were cultured for 4 days in the presence of RANKL (10 ng/ml) and M-CSF (30 ng/ml) with either the vehicle (prethanol) or the indicated concentration of BSE. Multinucleated osteoclasts were visualised using TRAP staining. (b) TRAP+ MNCs were counted (left panel) and TRAP activity was measured (right panel). ^###^*P* < 0.001 (versus the control); ^*∗*^*P* < 0.05; ^*∗∗∗*^*P* < 0.001 (versus the RANKL-treated group). (c) The effect of BSE on the viability of BMMs was evaluated using the CCK-8 assay. Data are expressed as mean ± SD and are representative of at least three experiments.

**Figure 2:**
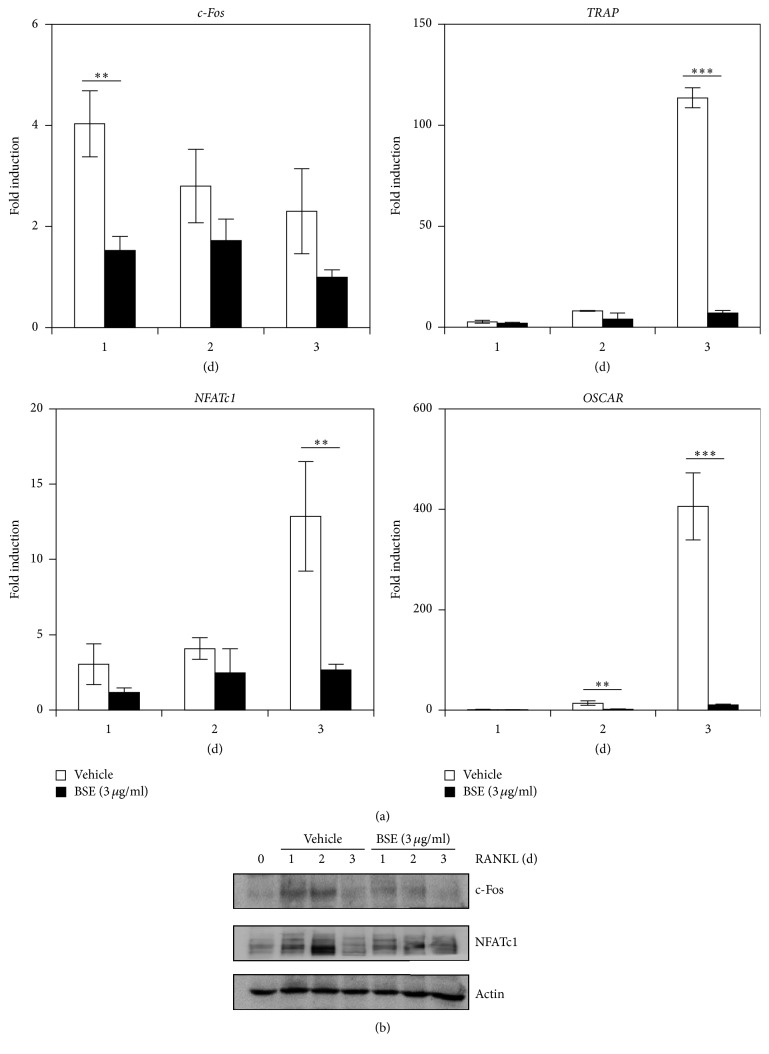
BSE inhibits the RANKL-mediated expression level of c-Fos/NFATc1. (a) The BMMs were stimulated with RANKL (10 ng/ml) and M-CSF (30 ng/ml) in the presence or absence of BSE (3 *μ*g/ml) for the indicated times. Total RNA was then isolated using TRIzol reagent, and the mRNA expression levels were evaluated using real-time PCR. GAPDH was used as the internal control. ^*∗∗*^*P* < 0.01; ^*∗∗∗*^*P* < 0.001 (versus the vehicle control). (b) The effect of BSE on the protein expression level of RANKL-induced transcription factors was evaluated using Western blot analysis. Actin was used as the internal control. Data are representative of at least three experiments.

**Figure 3:**
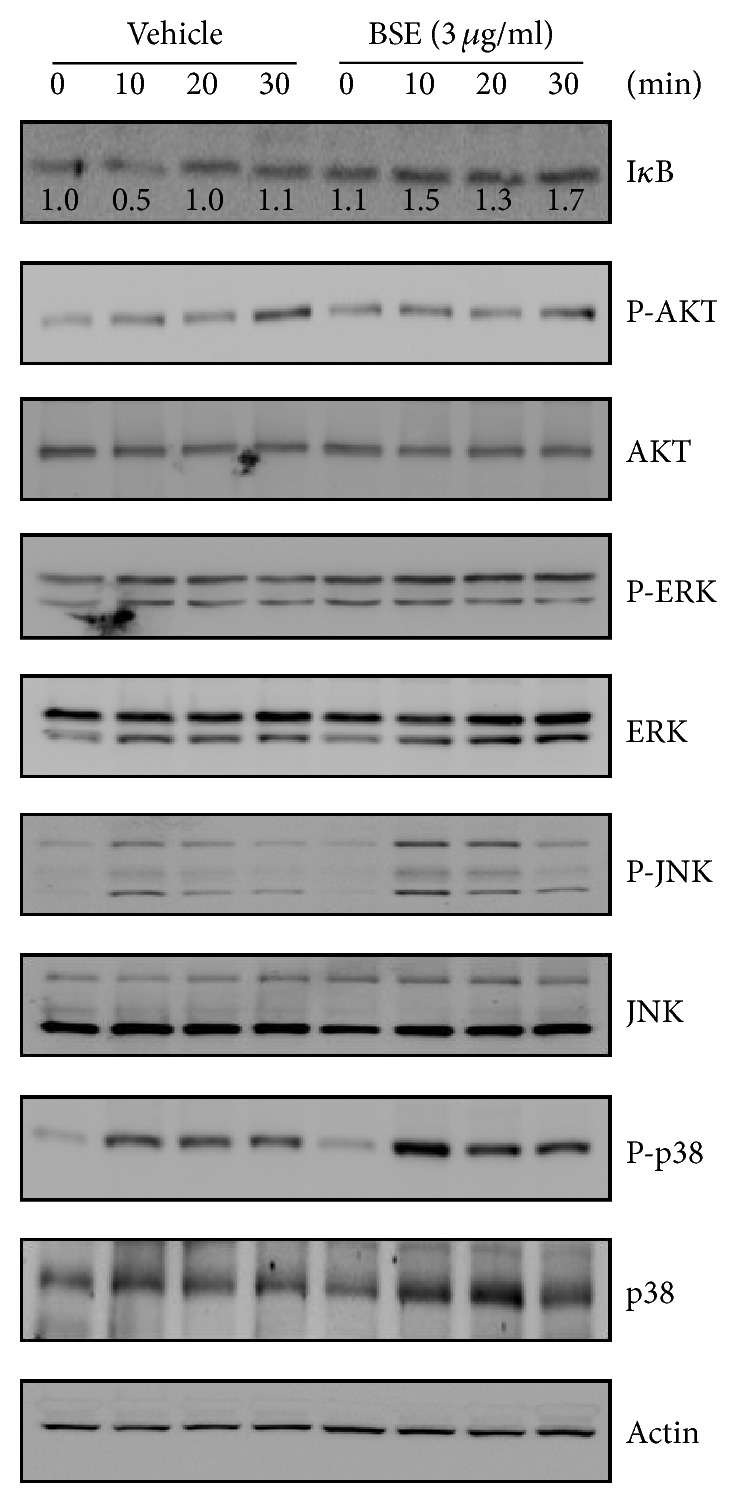
BSE attenuates RANKL-induced degradation of I*κ*B signaling molecules. In the condition of serum starvation for 1 hr, the BMMs were pretreated with or without BSE (3 *μ*g/ml) for 1 hr prior to RANKL stimulation (10 ng/ml) at the indicated time periods. Then, the expression levels of the signaling molecules were evaluated using Western blot analysis. The indicated densitometric values were obtained using Multi Gauge version 3 software. One representative result obtained from three independent experiments yielding similar results is shown.

**Figure 4:**
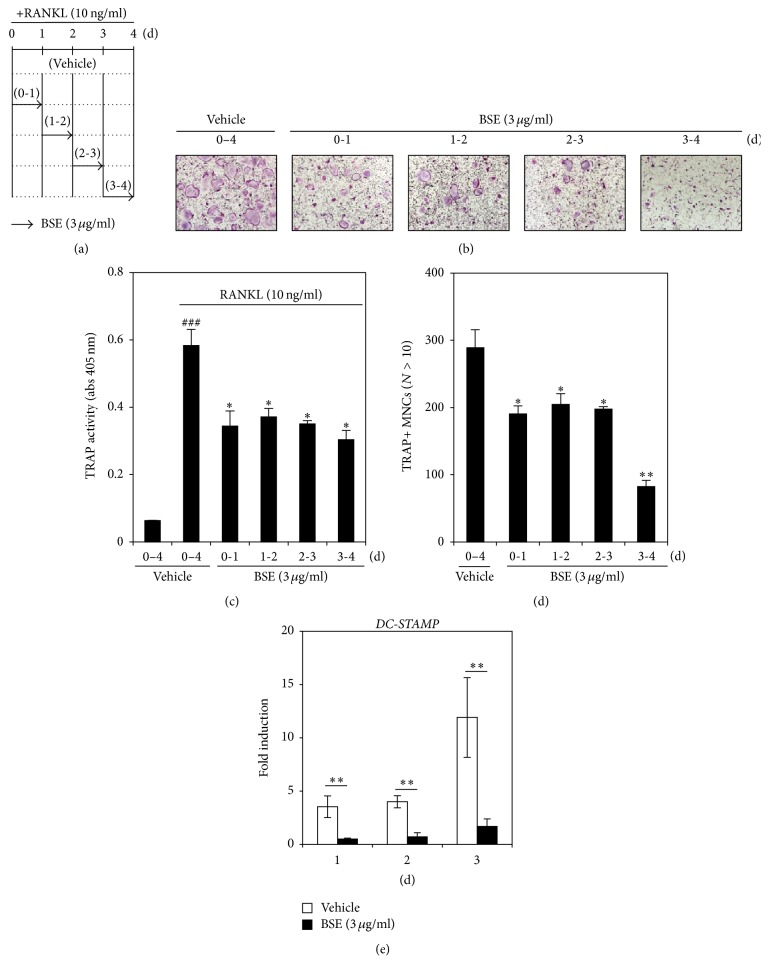
BSE also inhibits RANKL-mediated cell fusion during osteoclastogenesis. (a) Based on the exposure schedule, the BMMs were cultured with BSE (3 ug/ml) for various times periods (the indicated black arrow) in the presence of M-CSF (30 ng/ml) and RANKL (10 ng/ml). (b) After the BMMs differentiated into osteoclasts (as described in (a)), the cells were fixed, permeabilised, and stained with TRAP. TRAP+ MNCs formation was photographed under a light microscope. Each exposure period of BSE was indicated as “0–4” for the vehicle. “0-1” for 0-1 day, “1-2” for 1-2 days, “2-3” for 2-3 days, and “3-4” for 3-4 days. (c) TRAP activity was measured at 405 nm. ^###^*P* < 0.001 (versus the control); ^*∗*^*P* < 0.05 (versus the RANKL-treated control). (d) The number of TRAP+ MNCs (nuclei > 10) was counted. ^*∗*^*P* < 0.05; ^*∗∗*^*P* < 0.01 (versus the RANKL-treated group). (e) The effect of BSE on the mRNA expression of DC-STAMP was analysed using real-time PCR, as described in Figure 2(a). HPRT was used as the internal control. White column (vehicle-treated); black column (3 *μ*g/ml BSE-treated), ^*∗∗*^*P* < 0.01 (versus the vehicle control). Data are representative of at least three experiments.

**Figure 5:**
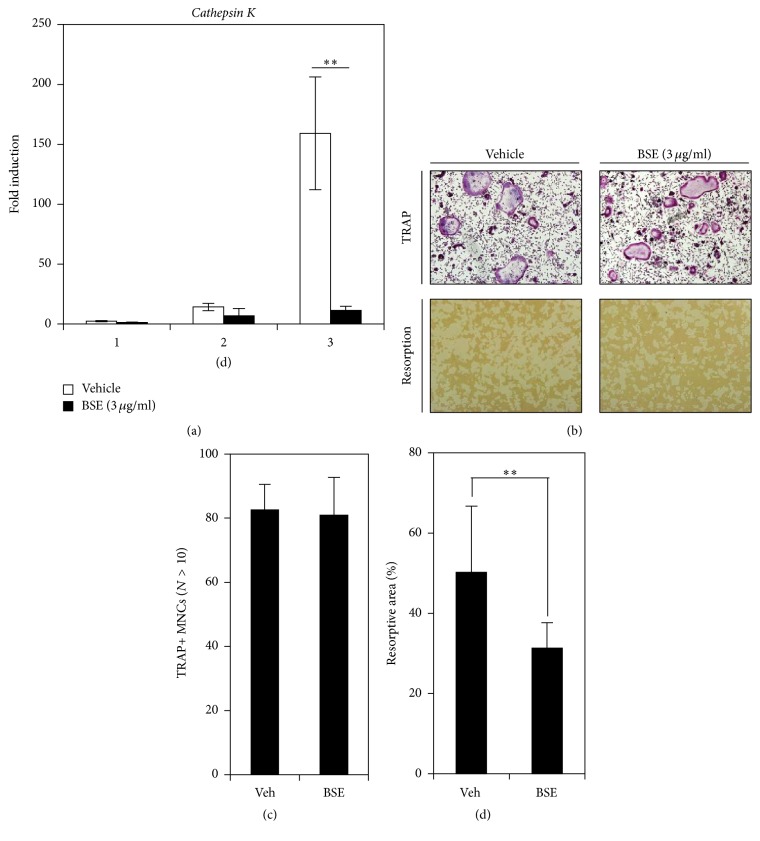
BSE impairs the osteoclastic function of giant multinucleated cells. (a) The mRNA expression of cathepsin K was evaluated during osteoclast differentiation in the absence or presence of BSE (3 *μ*g/ml) using real-time PCR. The relative fold change of the mRNA expression level is presented in comparison to the control (no RANKL-treated). HPRT was used as the internal control. ^*∗∗*^*P* < 0.01 (versus the vehicle control). (b) The mature osteoclasts were plated on bone biomimetic synthetic surface and treated for 24 hrs with BSE (3 *μ*g/ml). The cells were fixed, permeabilised, and stained with TRAP. TRAP+ MNCs formation was visualised under a light microscope (top images). The resorption areas were removed from the cells and photographed under a light microscope (bottom images). (c-d) The form (as visualised in (b)) was counted as the number of TRAP+ MNCs (nuclei > 10; (c)), and the resorptive areas (%) were quantified using the ImageJ program (d). ^*∗∗*^*P* < 0.01 (versus the vehicle control). One representative result achieved from three independent experiments yielding similar results is shown.

All quantitative values are presented as mean ± standard deviation. Each experiment in triplicate was performed three to five times, and Figures 1–5 show the results from one representative experiment. Statistical differences were analysed using Student's *t*-test, and a value of *P* < 0.05 was considered significant.

## 3. Results

### 3.1. BSE Inhibits RANKL-Induced Osteoclast Differentiation

To determine the effect of BSE on RANKL-mediated osteoclastogenesis, the BMMs were incubated with different concentrations of BSE followed by RANKL (10 ng/ml) treatment. The BMMs induced numerous TRAP-positive multinucleated osteoclast cells (TRAP+ MNCs) by RANKL in the control group (vehicle treatment), but BSE attenuated the formation of TRAP+ MNCs in a dose-dependent manner (Figure 1(a)). The inhibitory effect was confirmed by counting the number of TRAP+ MNCs (Figure 1(b); left panel) and measuring TRAP activity (Figure 1(b); right panel). Since the cellular cytotoxicity of BSE in the survival of the BMMs could affect RANKL-induced osteoclast differentiation, its effect was examined using the CCK-8 assay. As shown in Figure 1(c), no cytotoxicity of BSE was observed at the indicated dose. These results show that BSE significantly inhibited RANKL-mediated osteoclast differentiation without apparent cytotoxicity.

### 3.2. BSE Attenuates RANKL-Induced Expression of c-Fos and NFATc1 during Osteoclastogenesis

The inhibitory effect of BSE on osteoclast differentiation was examined by evaluating the expression level of several osteoclastogenesis-associated genes, including transcriptional factors. As shown in Figure 2(a), the mRNA expression levels of osteoclastogenesis-related transcription factors, such as c-Fos and NFATc1, were induced by RANKL, but these inductions were significantly inhibited by treatment with BSE. In addition, BSE also strongly attenuated the mRNA induction of c-Fos/NFATc1-dependent molecules, such as TRAP and OSCAR. Western blot analysis further revealed that the RANKL-induced translational expression of both c-Fos and NFATc1 was strongly inhibited by treatment with BSE (Figure 2(b)). Taken together, these results suggest that the antiosteoclastogenesis activity of BSE could arise from its potential to inhibit the expression of c-Fos/NFATc1, the early-stage transcription factor that is required for osteoclast differentiation.

### 3.3. BSE Contributes to RANKL-Mediated NF-*κ*B/I*κ*B Signaling Pathways

To clarify the mode of antiosteoclastic action by BSE, we investigated whether or not BSE could affect the activation of the RANKL-mediated signaling molecules associated with the regulation of c-Fos/NFATc1 expression, which are master transcription factors required for osteoclast differentiation. As shown in Figure 3, RANKL stimulated degradation of I*κ*B and the activation of RAC-Alpha Serine/Threonine-Protein Kinase (AKT) and MAP kinases, including extracellular signal-regulated kinase (ERK), c-Jun N-terminal kinase (JNK), and p38, but BSE only blocked the RANKL-induced degradation of I*κ*B. These results demonstrate that attenuation of I*κ*B degradation could be involved in the antiosteoclastogenic action of BSE.

### 3.4. BSE Inhibits Osteoclast Differentiation in the Late Stage Associated with Cell Fusion as well as in the Early Stages

To better understand when BSE inhibits osteoclast differentiation, we examined the antiosteoclastogenic activity of BSE by treating the cells at four time points, as shown in Figure 4(a). Treatment with BSE for 24 hrs moderately inhibited the RANKL-induced formation of TRAP+ MNCs in the early-to-late stages of osteoclastogenesis (Figure 4(b)). Moreover, TRAP activity was also attenuated by the addition of BSE (Figure 4(c)). Interestingly, the presence of BSE (3 *μ*g/ml) from day 3 to day 4 dramatically repressed TRAP+ MNCs formation with >10 nuclei giant osteoclasts (Figure 4(b), 3-4 d) and reduced the number of fused cells (Figure 4(d)). We confirmed the inhibitory effect of BSE on the monocyte TRAP+ cells into giant multinucleated osteoclasts by evaluating the mRNA expression level of DC-STAMP, which is an essential factor for osteoclast fusion. BSE strongly inhibited the RANKL-induced mRNA expression of DC-STAMP (Figure 4(e)). These results indicate that the antiosteoclastogenic effect of BSE could be due to its potential to inhibit multistep response in the early, middle, and late stages of osteoclast differentiation.

### 3.5. BSE Prevents the Bone-Resorbing Function of Mature Osteoclasts

Next, to investigate whether BSE has the potential to inhibit the survival and the bone-resorbing activity of mature osteoclasts, we performed resorption-related gene expression, mature osteoclast counting, and a bone pit formation assay. As shown in Figure 5(a), BSE significantly inhibited the RANKL-mediated mRNA induction of cathepsin K, which plays a role in bone resorption. We then confirmed the effect of BSE on the RANKL-induced bone resorptive function of mature osteoclasts in a coculture system of BMMs and primary osteoblast cells. When the purified mature osteoclasts from the coculture were replated on a bone biomimetic synthetic surface and cultured with/without BSE for 1 day, no significant difference was observed between the BSE-treated cells and the control group in terms of TRAP+ MNCs formation (Figure 5(b); upper panel) and the number of TRAP+ MNCs (Figure 5(c)). However, the addition of BSE strongly inhibited the areas of resorption formation (Figure 5(b); bottom panel) as measured using the resorbing bone pit assay (Figure 5(d)). These results revealed that BSE could prevent the bone resorptive function of mature osteoclasts without any alterations in the cell survival of the giant multinucleated osteoclasts.

## 4. Discussion

Antiosteoclastic agents have become the therapeutic mainstay for treating osteoporosis. However, the most common antiresorptive agents, such as bisphosphonates, also carry the risk of side effects, such as bisphosphonate-mediated osteonecrosis of the jaw [30] and atypical femoral fractures [31]. Consequently, the use of antiosteoclastic agents is limited due to concerns about their long-term safety. Therefore, new, safe therapeutic agents are urgently needed for the long-term management of bone disease.

Plant-based natural products have traditionally yielded a variety of therapeutic agents. Generally, healthy nutrients or foods with pharmaceutical properties are both effective and safe for the long-term administration of a variety of disorders. Recently, studies have attempted to identify natural products or healthy foods that can prevent and/or treat osteoporosis with minimal adverse effects [32].

As a major food crop for humans, barley is the second most commonly consumed grain in Korea, and it is recognised as a safe and healthy food to consume. Several studies have shown that BSE and its components exhibit antioxidant activities, decrease blood glucose and cholesterol levels, and protect against liver injury [33–36]. Nonetheless, the antiresorptive activity and mode of action of BS in bone metabolic diseases have not been revealed. This current study is the first to report on the antiosteoclastogenesis and inhibition of bone-resorbing activity of BSE.

The differentiation of osteoclasts from hematopoietic stem cells in bone marrow is specifically regulated by RANKL [37]. RANKL signaling triggers osteoclast formation, which is considered to be an important target for preventing pathological bone loss. In this study, BSE attenuated the RANKL-related differentiation of BMMs into osteoclasts in a dose-dependent manner without any cytotoxicity in concentrations up to 10 *μ*g/ml.

RANKL stimulates transcription factors, such as c-Fos and NFATc1, during osteoclast differentiation. As activator protein-1 (AP-1) family members, c-Fos and NFATc1 play a major role in the regulation of molecules for osteoclast differentiation. An important role for c-Fos in the process of osteoclast differentiation has been clarified in c-Fos knockout mice [38]. The c-Fos-deficient mice had osteopetrosis due to a cell-autonomous defect in osteoclast differentiation [39]. Furthermore, Takayanagi et al. [40] reported that NFATc1-deficient embryonic stem cells do not differentiate into mature osteoclasts, even in the presence of RANKL. As major transcription factors, c-Fos and NFATc1 are also functionally linked together. Particularly, c-Fos is essential for RANKL-induced expression of NFATc1. c-Fos is expressed in the early stages of osteoclast differentiation, and it regulates NFATc1 gene expression by binding to the promoter region of NFATc1 [40]. NFATc1 is expressed in the middle or late stages of osteoclastogenesis, and it regulates osteoclast-mediated genes, such as TRAP, and OSCAR [9, 41]. Therefore, c-Fos and NFATc1 are master regulators for RANKL-induced osteoclast differentiation. In this study, two transcription factors such as c-Fos and NFATc1 inhibited the expression of the transcriptional and translational levels by BSE treatment during osteoclast differentiation. In addition, the inhibitory effect of BSE via downregulation of c-Fos and NFATc1 was confirmed by evaluating the transcriptional expression levels of c-Fos/NFATc1-dependent genes, such as TRAP and OSCAR. These results suggest that c-Fos/NFATc1, as a main transcriptional marker of osteoclastogenesis, is involved in BSE's inhibitory effect on osteoclast differentiation.

The binding of RANKL to its receptor RANK activates various signaling pathways, including NF-*κ*B, PI3K/AKT, and MAP kinases, consisting of p38, ERK, and JNK, in the early stage of osteoclastogenesis [37, 42]. It is known that the expression of c-Fos/NFATc1 requires assembly of NF-*κ*B, PI3K/AKT, and MAP kinase signaling [40, 43–45]. In this present study, the pathways for the PI3K/AKT and MAP kinases were not affected by BSE. However, BSE prevented the alternation of the RANKL-induced degradation of I*κ*B signaling molecules, which leads to NF-*κ*B activation. I*κ*B is a member of a family of cellular proteins that function to inhibit NF-*κ*B molecules. I*κ*B inhibits NF-*κ*B by masking the nuclear localisation signals of NF-*κ*B proteins and keeping them sequestered in an in-active state in the cytoplasm [46]. In IKK-knockout mice, the upstream I*κ*B die at birth as a result of severe epidermal defects; however, analysis of the embryonic osteoclasts (E18.5 days postcoitus) revealed reduced numbers of multinucleated osteoclasts with altered morphology [47]. Additionally, NF-*κ*B p50/p52 double knockout mice display defects of osteoclast differentiation and severe osteopetrosis because c-Fos/NFATc1 was not induced by RANKL [48]. These reports indicated that the NF-*κ*B/I*κ*B signaling pathway could have the potential to regulate osteoclast differentiation through c-Fos/NFATc1 expression. In this present study, the inhibitory effect of BSE on osteoclastogenesis could result from its potential ability to attenuate the NF-*κ*B/I*κ*B-c-fos/NFATc1 signaling axis at the early stage of osteoclast differentiation.

Osteoclast differentiation is a multistep progressive process that involves cell proliferation, commitment, migration, fusion, and maturation [49, 50]. Monocyte/macrophage precursors derived from hematopoietic stem cells in the bone marrow become preosteoclasts that then migrate and fuse to form giant multinucleated osteoclasts. In this regard, we next investigated whether the inhibitory effect of BSE affected some of the stages of osteoclast differentiation. To determine this, we reevaluated the antiosteoclastogenic activity of BSE by treating cells at each osteoclastogenesis stage. Interestingly, the TRAP+ MNCs formation by BSE was suppressed during the early, middle, and late stages of osteoclast differentiation.

Cell-cell fusion is essential for the formation and maturation of multinucleated osteoclasts; this process involves cellular fusion molecules, such as DC-STAMP. In osteoclastogenesis, DC-STAMP has been reported to be essential for cell-to-cell fusion of preosteoclasts, and it is preferentially expressed in the late stage [51–53]. Osteoclasts derived from DC-STAMP-deficient mice were found to abrogate multinucleated osteoclast formation and increase bone mass [54]. Additionally, DC-STAMP contains multiple NFATc1 binding sites in promoter regions, and it is regulated by NFATc1 transcription factor [55]. As mentioned earlier, we found that the mRNA expression levels of DC-STAMP and NFATc1 were inhibited by BSE during osteoclast differentiation. Taken together, these results suggest that BSE with antiosteoclastogenic activity is involved in osteoclast differentiation from the initial phase of osteoclastogenesis to the terminal phase.

Bone resorption is a unique function of osteoclasts, and it plays a central role in the formation of the skeleton and the regulation of its mass, which is involved in the matrix degradation of the organic and inorganic phases of bone [56]. In this progression, active proteases released from osteoclasts into the resorption lacunae are known to be involved in matrix degradation, and it has been reported that cysteine proteinases play a vital role in this process [57, 58]. Cathepsin K is a type of lysosomal cysteine protease, such as a proteolytic enzyme, and it is abundantly expressed in mature osteoclasts. It can degrade the protein components of the demineralised bone matrix, most notably type-1 collagen [59–61]. In addition, a study of cathepsin K-deficient mice found impaired bone loss via reduction of bone resorption and an increased bone formation rate in comparison to the control [62, 63]. RANKL-mediated cathepsin K expression has been shown to be regulated by NFATc1 [64]. Thus, induction of cathepsin K by NFATc1 is responsible for the degradation of the collagen matrix by osteoclasts. In our present study, the presence of BSE was associated with inhibition of the cathepsin K expression level and the anti-bone-resorbing activity of mature osteoclasts.

## 5. Conclusions

To the best of our knowledge, this is the first study to have shown the potential of a natural food, such as BSE, to inhibit osteoclast differentiation and bone-resorbing activity in the early-to-late stages of osteoclastogenesis, although additional experiments are needed to substantiate the identification of the pharmaceutical components in BSE for antiosteoclastogenic activity. BSE inhibits RANKL-induced osteoclastogenic activity by preventing I*κ*B degradation and c-Fos/NFATc1 expression. Consequently, the alteration of I*κ*B/c-Fos/NFATc1 could lead to the decreased expression of the genes required for bone-resorbing activity and cell fusion, such as DC-STAMP and cathepsin K. Moreover, BSE prevented the bone-resorbing activity of mature osteoclasts. Finally, our results suggest that the potential antiresorptive property of BSE might be developed as a functional food and pharmacological agent to improve bone health and to treat osteoclast-mediated bone metabolic disorders, including osteoporosis.

## Figures and Tables

**Table 1:** The primer sequences used in this study.

Target gene	Forward primer (5′–3′)	Reverse primer (5′–3′)
*c-Fos*	CCAGTCAAGAGCATCAGCAA	AAGTAGTGCAGCCCGGAGTA
*NFATc1*	GGGTCAGTGTGACCGAAGAT	GGAAGTCAGAAGTGGGTGGA
*TRAP*	GATGACTTTGCCAGTCAGCA	ACATAGCCCACACCGTTCTC
*OSCAR*	AGGGAAACCTCATCCGTTTG	GAGCCGGAAATAAGGCACAG
*DC-STAMP*	CCAAGGAGTCGTCCATGATT	GGCTGCTTTGATCGTTTCTC
*Cathepsin K*	GGCCAACTCAAGAAGAAAAC	GTGCTTGCTTCCCTTCTGG
*GAPDH*	ACCACAGTCCATGCCATCAC	TCCACCACCCTGTTGCTGTA
*HPRT1*	TGCTCGAGATGTCATGAAGG	AGAGGTCCTTTTCACCAGCA
